# Preliminary examination of the effects of an early parenting intervention on amygdala-orbitofrontal cortex resting-state functional connectivity among high-risk children: A randomized clinical trial

**DOI:** 10.1017/S0954579423001669

**Published:** 2024-01-22

**Authors:** Marta Korom, Emilio A. Valadez, Nim Tottenham, Mary Dozier, Jeffrey M. Spielberg

**Affiliations:** 1 Department of Psychological and Brain Sciences, University of Delaware, Newark, DE, USA; 2 Department of Human Development and Quantitative Methodology, University of Maryland, College Park, MD, USA; 3 Department of Psychology, Columbia University, New York, NY, USA

**Keywords:** Parenting intervention, imaging, resting-state, amygdala↔OFC functional connectivity, emotion regulation, adversity

## Abstract

We examined the long-term causal effects of an evidence-based parenting program delivered in infancy on children’s emotion regulation and resting-state functional connectivity (rs-fc) during middle childhood. Families were referred to the study by Child Protective Services (CPS) as part of a diversion from a foster care program. A low-risk group of families was also recruited. CPS-involved families were randomly assigned to receive the target (Attachment and Biobehavioral Catch-up, ABC) or a control intervention (Developmental Education for Families, DEF) before infants turned 2. Both interventions were home-based, manualized, and 10-sessions long. During middle childhood, children underwent a 6-min resting-state functional MRI scan. Amygdala seed-based rs-fc analysis was completed with intervention group as the group-level predictor of interest. Fifty-seven children (*N*_ABC_ = 21; *N*_DEF_ = 17; *N*_COMP_ = 19; *M*_age_ = 10.02 years, range = 8.08–12.14) were scanned successfully. The DEF group evidenced negative left amygdala↔OFC connectivity, whereas connectivity was near zero in the ABC and comparison groups (ABCvsDEF: Cohen’s *d* = 1.17). ABC may enhance high-risk children’s regulatory neurobiology outcomes ∼8 years after the intervention was completed.

## Introduction

In the first years of life, when infants are dependent on their parents for mobility, food, and safety, the developing infant’s brain undergoes a complex neurobiological transformation (Dobbing & Sands, 1973). Central to this transformation are early attachment relationships that buffer the infant from environmental threats and scaffold the organization of neural connections into small- and large-scale brain networks (Hostinar et al., 2014). Sensitive and nurturing caregiving builds the foundation for optimal neural development, especially in regions involved in emotion- and behavior-regulation (Korom & Dozier, 2021; Bourne et al., 2022; Tottenham, 2017). Conversely, insensitive care (e.g., neglect, abuse) threatens healthy attachment relationships and early neurodevelopment of the infant (Callaghan & Tottenham, 2016a). Such inadequate early care can undermine the optimal development of the infant’s regulatory neurobiology, potentially leading to long-term emotion-*dys*regulation (Callaghan & Tottenham, 2016b; Milojevich et al., 2020).

The connectome shows a complex developmental pattern, with sensory regions becoming mature early in development, whereas maturation is prolonged in regions supporting higher-order cognitive and socio-emotional functioning (Hensch & Bilimoria, 2012). One region with a protracted period of development is the orbitofrontal cortex (OFC), which partially overlaps with the ventromedial prefrontal cortex (vmPFC) (Tottenham, 2020). All regions of the OFC exhibit strong structural and functional connections with the amygdala and play an important role in emotional health. Specifically, the OFC is key to subjective socio-affective processing (Bechara et al., 2000; Rudebeck et al., 2013) due to its role in integrating reward value with visceral and sensory information (visual, gustatory, somatosensory, and olfactory). The medial OFC, in particular, assigns positive subjective value to rewarding experiences, whereas the lateral OFC represents punishment, non-reward, and subjectively aversive experiences (Kringelbach, 2005; Rolls et al., 2020). Animal models have also shown that aberrant lateral OFC activation is associated with poor regulation of fear-related behaviors (Shih & Chang, 2021) and fear learning (Sun & Chang, 2022). In line with animal models, damage to the OFC in humans has been associated with impaired subjective appraisals of hedonic experiences, poorly reinforced emotional and behavioral learning (Rolls & Grabenhorst, 2008), and increased vulnerability to behavioral dysregulation and emotional disorders, such as depression (Rolls et al., 2022). Taken together, growing evidence suggests that the OFC plays an important role in the appraisal of both appetitive and aversive experiences and the regulation of emotion and behavior, reflecting a potential pathway through which both rewarding and non-rewarding interactions with caregivers early in life can affect children’s brain development.

The amygdala also plays a central role in affect, including directing attention toward relevant stimuli (e.g., threat) and learning connections between stimuli and associated contextual information (Hartley & Lee, 2015). The protracted development of amygdala↔OFC connectivity serves a critical evolutionary function, allowing the developing brain to maintain neuroplasticity while learning to navigate complex social and emotional environments (Tottenham, 2020). Although such plasticity allows for extensive adaptation to the environment, it can also be a source of vulnerability through which early-life stressors negatively impact development.

Emerging work suggests that the maturational pattern of the amygdala↔OFC resting-state functional connectivity (rs-fc) shows weak functional coupling during childhood and becomes increasingly positive as children mature (Gabard-Durnam et al., 2018; Hahn et al., 2011; Thijssen et al., 2021). This pattern of development is hypothesized to allow the OFC increasing capacity to regulate amygdala-mediated arousal to emotionally salient cues, especially cues perceived as potentially threatening (LeDoux, 2000; Wood & Grafman, 2003). In typically developing children, consistent positive coupling emerges in late middle childhood/early adolescence (Gabard-Durnam et al., 2014; Qin et al., 2012), which may indicate that amygdala↔OFC coupling increasingly supports independent regulation over development (Gabard-Durnam et al., 2014).

To help children regulate their emotions effectively during this period of neurobiological immaturity, attachment figures may fulfill a key part of the regulatory role for the child until the PFC is mature enough to fulfill this role independently of a co-regulator (Gee, 2016; Tottenham, 2020). Both the structural and functional development of the OFC and the amygdala are susceptible to variations in the quality of early care (Cheng et al., 2021; Hanson et al., 2013). Sensitive and nurturing care supports infants’ survival when, unlike adults, infants are unable to mobilize an effective fight-or-flight response. Parents can buffer the infant’s emotional and physiological reactivity to stress (Evans & Porter, 2009; Hostinar et al., 2014) and act as an external co-regulator of emotion and behavior for the infant (Hofer, 2010). For instance, supportive care reduces cortisol reactivity and increases cortisol recovery in infants (Gunnar & Donzella, 2002) and can buffer conditioned fear learning (Egliston & Rapee, 2007).

The absence of a nurturing parent can impair self-regulation, thus increasing long-term vulnerability to emotional disorders (Gee, 2016). In particular, the absence of emotionally attuned, responsive, and nurturing parenting is associated with atypical amygdala↔OFC connectivity in children between 8 and 10 years of age, but the direction of this effect has been inconsistent across studies. For example, when comparing those exposed to abuse, neglect, and/or violence to typically developing youth, positive (Cheng et al., 2021) and near zero (Thomason et al., 2015) amygdala↔OFC rs-fc have been observed in children with a history of adversity. Moreover, others have found no significant differences between these groups in amygdala↔OFC coupling (Nooner et al., 2013; Saxbe et al., 2018). Cheng and colleagues (2021) found negative coupling between amygdala and other PFC regions in a group of adversity-exposed adolescents, and Fan and colleagues (2014) found negative associations between emotional abuse and amygdala↔OFC rs-fc in adolescents. These results suggest that positive, negative, and near zero rs-fc between amygdala and PFC are plausible consequences of early insensitive care. These inconsistencies may be driven by the heterogeneity of developmental periods (Jalbrzikowski et al., 2017) and PFC areas examined, or by the variability in the type or timing of adverse experiences. Regardless, a key limitation of these studies is that the study designs have not allowed causal inferences about the effect of the quality of early care on children’s brain development and behavior.

In cases of insensitive care, evidence-based early parenting interventions are crucial to prevent altered neurodevelopment and support long-term emotion circuitry development (Valadez et al., 2020, 2023). The most direct way to determine the causal effects of early parental care on resting-state connectome is via a randomized clinical trial (RCT) of an efficacious treatment that follows children’s development over time. One such intervention is Attachment and Biobehavioral Catch-up (ABC), a 10-session program for the parents of infants that is delivered in families’ homes (Dozier & Bernard, 2019). ABC is typically compared to a control intervention called Developmental Education for Families (DEF) (Brooks-Gunn et al., 1993). DEF is also a manualized, 10-session program delivered in the home, but it differs from ABC in its intervention targets. ABC parent coaches provide in-the-moment comments about parental behavior while the parent is interacting with their infant. ABC specifically seeks to (i) enhance contingently responsive care when the infant is not distressed, (ii) enhance nurturing care when the infant is distressed, and (iii) reduce frightening or harsh behaviors at all times. These target behaviors are particularly important for infants at risk for early caregiving adversities, including a history of Child Protective Services (CPS) involvement or institutional care (Dozier & Bernard, 2019). Conversely, DEF targets motor and language development, but not responsive parental behaviors. In multiple RCTs, ABC has been found to causally enhance parental sensitivity (Bick & Dozier, 2013; Hepworth et al., 2021; Yarger et al., 2016), children’s attachment security (Bernard et al., 2012), executive functioning (Korom et al., 2021; Lind et al., 2017), emotion regulation (Labella et al., 2020), increase neural activation associated with representations of maternal cues (Valadez et al., 2020), and top-down regulation of fearful faces relative to DEF (Valadez et al., 2023). However, little is known about how early enhanced care shapes high-risk children’s rs-fc development in circuitry supporting affect. Considering that recurring early experiences (e.g., experiences of threat or safety) shape the development of children’s resting-state functional architecture (Gabard-Durnam et al., 2016), it is critical to understand how enhanced care can support neurobiological adaptation and emotion regulation in children at risk for caregiving adversities.

In this preliminary study, we leveraged an RCT of ABC to understand the long-term causal effects of this efficacious program on children’s resting-state functional connectivity (rs-fc) approximately 8 years after the intervention was completed. Following allegations of suboptimal care (e.g., neglect, homelessness, and possible abuse) that resulted in CPS involvement, participating mothers (with infants younger than 2 years old) were randomly assigned to receive ABC or DEF. Families were followed longitudinally, and children returned to the lab at ages 8, 9, and 10 years. We also recruited a group of typically developing 8-year-olds from the community to serve as a low-risk comparison group. A subset of the full sample was recruited for this MRI sub-study. Our primary hypothesis aimed to examine the causal effect of intervention assignment on amygdala↔OFC rs-fc outcomes. We hypothesized that children in the ABC group would show significantly different amygdala↔OFC rs-fc coupling than children in the DEF group. We also hypothesized that the amygdala↔OFC rs-fc coupling in the low-risk group would be more similar to the ABC group than the DEF group. Finally, our secondary hypothesis posited that the observed amygdala↔OFC rs-fc would support better emotion regulation than that seen in the DEF group. Given our goal to investigate the longitudinal causal effect of an early parenting intervention on children’s neurobiology, the implementation of a between-person randomized clinical trial was necessary (Hernán & Robins, 2020; Rohrer & Murayama, 2023; Shadish & Sullivan, 2012). A within-person longitudinal study design was not feasible given the age of the participants at the time when the intervention was implemented (average post-intervention age = 19.2 months). Importantly, between-person experimental designs can inform us about average, causal post-intervention group differences if the randomization prior to the implementation of the intervention was successful.

## Method

### Study design and participants

Families were referred to the study by CPS as part of a diversion from foster care programs in a mid-Atlantic city between 2005 and 2008. Before infants turned 2 years old, families (*N* = 212) were randomly assigned to receive either ABC or DEF interventions (see Supplementary Figure 1 for CONSORT diagram). The participating child and/or other children in the family were considered at risk for receiving inadequate care due to educational or physical neglect, possible physical abuse, homelessness, or parental substance abuse. Using simple randomization, at a pre-intervention research visit, children were randomly assigned to one of two interventions (parallel design, 50%–50% allocation ratio using a random-number table). Allocation concealment was achieved by having the primary investigator generating the random number table, a research assistant enrolling participants, and a research coordinator allocating them to interventions. The intervention groups did not differ significantly from each other on demographic variables (age, race, minority status, see Table 1) or neuroendocrine (diurnal cortisol regulation) functioning (Bernard et al., 2012, 2015), which suggests that the randomization was successful. When the high-risk children turned 8 years old, 112 participated in three yearly middle-childhood assessments. Typically developing, low-risk children were also recruited when they were 8 years old to serve as low-risk comparisons. Supplemental NIMH funding was obtained for the purpose of MRI scanning a subset (*n* = 80) of the low- and high-risk groups around age 10 between 2015 and 2016. To increase the likelihood of a successful MRI scan, children were only invited to participate in the MRI sub-study if they successfully completed a prior EEG task, had an IQ higher than 70, and had no neurobiological disorders identified during the 8-year assessment. Exclusion criteria for this low-risk group included prior history of CPS-involvement, homelessness, and family history of drug abuse at the time of enrollment. Those assessing outcomes were blinded to intervention assignment.

The initial sample consisted of 27 children in the ABC group, 27 in the DEF group, and 26 children in the low-risk comparison group. Data for 23 of these children were excluded from analysis due to excessive head motion (>50% total volumes motion-scrubbed at .5 mm threshold), problems with data acquisition or image registration, or an incomplete resting-state scan (see Supplementary Figure 1 for CONSORT diagram). The analytic sample included: *N*_ABC_ = 21, *N*_DEF_ = 17, *N*_low-risk_ = 19. See Table 2 for further demographic information. The demographic information of the low-risk comparison group is available in Supplementary Table 2.

### Procedures and measures

Parents provided written consent and children assented to participating in the scanning study. Children underwent mock scanning to assess their comfort in the scanner, followed by an MRI scan. To contextualize the neuroimaging findings, parents were later asked to complete the Emotion Regulation Checklist (Shields & Cicchetti, 1997) in the laboratory to assess their children’s perceived emotion regulation outcomes. The average time between MRI and ERC was 5.75 months (MRI_age_ = 10.02 years; ERC_age_ = 10.5 years). The study protocol was approved by the Institutional Review Board of the University of Delaware, and all participants were treated in accordance with established ethical guidelines.

### Emotion regulation checklist

The ERC measures parents’ perception of their children’s ability to regulate emotions on a 4-point Likert scale. It has two subscales, and we used the emotion regulation (ERC-ER) subscale, which assesses children’s empathy, emotional expression, and regulatory capacity, with higher scores indicating better emotion regulation. The internal consistency of the ERC-ER was acceptable (Cronbach α = .638).

### Neuroimaging data

Imaging data were acquired on a 3-Tesla Siemens MAGNETOM Prisma scanner (Siemens, Erlangen, Germany), equipped with a 20-channel head coil. First, a whole-brain, high-resolution T1-weighted MPRAGE anatomical scan (magnetization-prepared rapid gradient-echo; in-plane resolution, 256 × 256; field of view, 256 mm; sagittal slices, 192 × 1 mm) was acquired. Next, a 5 min 51 s resting-state T2*-weighted echo-planar scan was acquired (176 volumes, 34 slices, TR = 2,000 ms, echo time = 30 ms, flip angle = 90°, voxel size = 3.125 × 3.125 × 4 mm), during which children were instructed to close their eyes but stay awake.

### Data processing and statistical analyses

#### Power analysis

In previous studies with similar populations, sample size, and methodology (e.g., Hardee et al., 2013), large effect sizes emerged for many analyses (*d* > 1). For analyses involving two groups, power to detect a large effect was .92, and power to detect a medium effect was .57. In our prior work, with the same group of children and methodology (Valadez et al., 2020, 2023) large effect sizes emerged.

#### MRI preprocessing and first-level analysis

Separate left and right amygdala masks were created via the FreeSurfer v.6 standard processing pipeline, and white matter and ventricular masks were created via FSL’s FAST, all of which were confirmed visually and were converted into each participant’s resting-state functional space. Resting-state fMRI data were pre-processed using Graph Theoretic GLM toolbox (GTG; https://www.nitrc.org/projects/metalab_gtg) v.0.5, which uses tools from the FSL toolbox. Preprocessing steps included motion correction (via McFlirt), second-order polynomial detrending, bandpass filtering (.01–.1 Hz), motion censoring (cutoff = .5 mm FD or 2.5 DVARS), and partialing of nuisance signals, including motion parameters (6 + original 6 *n* − 1 = 12), and the mean white matter, ventricular, and global signal. The squared versions of each of these parameters (except the *n* − 1 motion) were also included, along with the temporal derivatives of all signals, resulting in a total of 48 nuisance parameters.

The timeseries for each amygdala was then extracted (via the first principal component) and entered as a predictor into a first-level FEAT (along with the temporal derivative) for seed-based analyses, with the processed data from the GTG toolbox as the dependent variable. In FEAT, spatial smoothing (5 mm FWHM) was then applied and the resultant beta maps were registered to MNI 2009a Nonlinear Asymmetric 1 mm^3^ template.

#### Primary analyses (group-level MRI analyses):

Beta maps from the first-level analyses were entered as dependent variables into RANDOMISE (5,000 permutations), with the predictor of interest being the ABC vs. DEF comparison. Nuisance covariates included the number of usable volumes for each participant after motion censoring and four DVARS-based parameters, including the mean standard and slow-wave (SVARS) variants and their square. Threshold-free cluster enhancement was applied to the resultant beta map and correction for multiple comparisons was limited to voxels within the OFC. We used the Julich-Brain probabilistic atlas (Amunts et al., 2020) to create an OFC mask by adding together the maximum probability versions of all seven subregions of the OFC (i.e., Fo1 through Fo7) and binarized the result. To see the extent of the mask, see Supplementary Figure 2.

#### Secondary analyses

Mean betas were extracted from significant clusters for follow-up analyses in R v.3.6.1. To determine which group deviated from the typical pattern of development, mean betas for significant clusters identified via the RCT contrast were also extracted from the low-risk comparison group and *t*-tests were performed to compare each high-risk group to the low-risk group.

#### Exploratory analyses

To contextualize the results of the primary analyses, we completed planned exploratory analyses. In a multiple regression model, we examined whether intervention assignment interacted with the extracted cluster estimates in predicting the participants’ parent-reported emotion regulation outcomes. Covariates of no interest included age and sex. We chose not to include the time lag between the MRI scan and the completion of the ERC in the model because age and time lag were highly correlated (*r* = −.904, *p* < .001). By not including time lag in the mode, we avoided issues with multicollinearity and loss of statistical power due to having too many predictors given the modest sample size.

Additional analyses were completed without age and sex in the model.

#### Data sharing, intervention material sharing, and preregistration statement

The data used in this study are available on request from the corresponding author, MK. The data are not publicly available because: (1) we did not obtain permission from parents to share these data and (2) the small sample size and unique design might make it possible for participants to be identified. This study was not pre-registered. The clinical trial was pre-registered (https://clinicaltrials.gov/ct2/show/NCT02093052). The ABC manual is only available to those receiving training in the ABC model because the ABC intervention developers are concerned that implementation without training and supervision would not be true to the model and would therefore be ineffective.

## Results

### Demographics

ABC and DEF did not differ in age, parental educational background, income, or sex, ethnic, and racial distribution. There were significant differences in income and parental education between the high-risk groups and the low-risk group (ABC and DEF vs. low-risk comparison group), but the ABC and DEF groups did not differ from each other (See Table 2 and Supplementary Table 2).

### Imaging-related descriptive statistics

The number of usable volumes (following motion censoring) were not significantly associated with participants’ rs-fc (*r* = −.244, *p* = .139) or intervention group assignment (ABC vs. DEF: *t*(36) = .909, *p* = .369). The correlation matrix of study variables is available in Supplementary Table 1.

### Primary analyses (group-level MRI)

**Figure 1.**
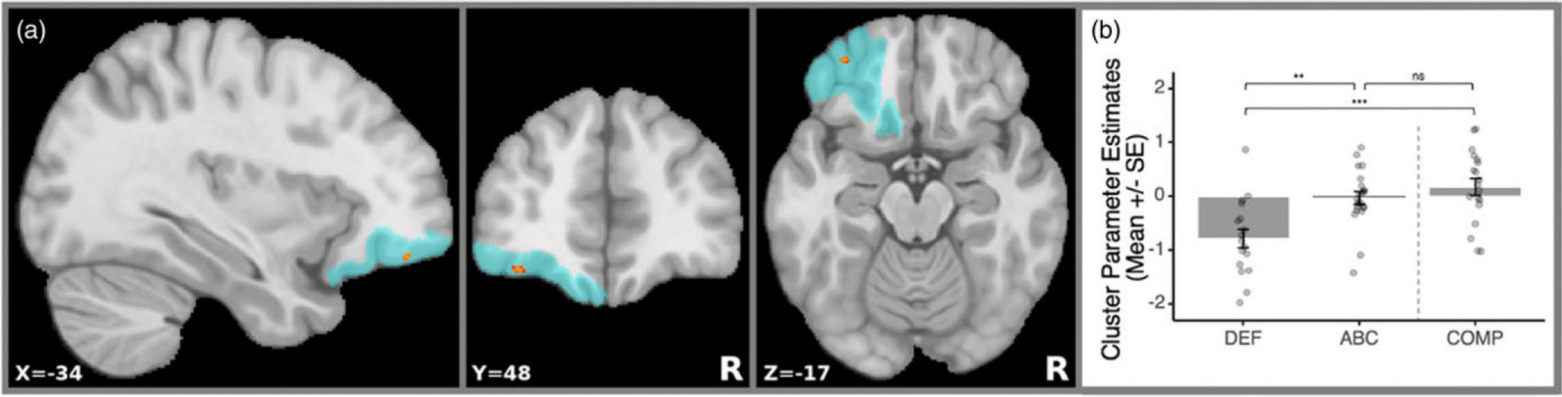
Intervention effects and cluster-masked mean estimates. (a) Location of the significant rs-fc cluster that survived multiple comparisons correction indicated with red. The blue area denotes the coverage of the Julich-Brain probabilistic OFC mask that was used. R=right. (b) Bar plot with overlaid distribution plot of cluster parameter estimates. The values correspond to the extracted rs-fc values at the OFC region where the significant intervention effect was found between the Attachment and Biobehavioral Catch-Up (ABC), Developmental Education for Families (DEF), and low-risk comparison (COMP) groups.

Separate analyses for ABC > DEF and ABC < DEF contrasts were completed with separate left and right amygdala seeds, and one cluster in the left anterior OFC (Figure 1a) survived multiple comparison corrections in the ABC > DEF contrast (cluster size = 26 voxels; center of gravity: *x* = −34; *y* = 48; *z* = −17; mean *p*-value = .038). Children in the ABC group had significantly less negative (near zero) left amygdala↔OFC functional connectivity than the DEF group (Figure 1b).

### Secondary analyses

Ancillary analyses revealed that ABC did not differ from the low-risk group (*b* = −.206, *t*(52) = −1.01, *p* = .319), but DEF did (*b* = −.945, *t*(52) = −4.357, *p* < .001), with the low-risk group having significantly less negative left amygdala↔OFC rs-fc than DEF.

### Exploratory analyses

There was no significant intervention effect on ERC (ABC vs. DEF; *b* = .005, *t*(34) = .028, *p* = .978). The main effects of amygdala↔OFC rs-fc and intervention group on ERC were also not significant (amygdala↔OFC rs-fc on ERC: *b* = .114, *t*(34) = .907, *p* = .371). There was a significant interaction between amygdala↔OFC rs-fc and intervention group in predicting ERC. Simple slopes analyses revealed that there was a positive association between amygdala↔OFC rs-fc and ERC in DEF but not in ABC (interaction effect size *b* = .587; see Supplementary Figure 3 and Table 3 for interaction and simple slope statistics). Age at scan and sex were included as covariates of no interest in these models.

## Discussion

The present study leveraged data collected as part of an RCT to identify the causal effects of an early parenting intervention on children’s amygdala↔OFC rs-fc. Our main hypothesis posited that the ABC and DEF groups would show significantly different amygdala↔OFC rs-fc, and that children in the ABC group would be more similar to the low-risk group than the DEF group. In line with our hypotheses, coupling in the ABC group was significantly different from the DEF group, but ABC did not differ significantly from the low-risk control group. Specifically, the DEF group showed significantly more negative amygdala↔OFC coupling than the ABC or the low-risk groups. Children in the ABC and low-risk groups had near-zero amygdala↔OFC coupling. Considering that amygdala↔OFC coupling becomes increasingly more positive as children develop (Gabard-Durnam et al., 2014; Qin et al., 2012), the negative rs-fc displayed in the active control intervention group (DEF) may suggest atypical amygdala↔OFC coupling during middle childhood or a form of adaptation to the low-quality early caregiving environment. Moreover, these group differences in connectivity appear to be causally driven by the intervention, given the random assignment to one of the two intervention groups. Thus, the ABC intervention may support typical amygdala↔OFC connectivity outcomes, which is consistent with our prior work showing that CPS-involved, foster, and internationally adopted children who received the ABC intervention have better emotion regulation during early childhood (Labella et al., 2020), as well as better inhibitory control (Korom et al., 2021) and social-emotional competence (Lind et al., 2021) relative to children in the control intervention group during middle childhood.

To contextualize our neuroimaging findings, we completed exploratory moderation analyses using parent-reported data on participants’ emotion regulation. Specifically, we explored the interaction between intervention assignment and amygdala↔OFC functional coupling in predicting children’s emotion regulation capacity. We found a significant interaction term, such that a positive association was found between children’s rs-fc and parent-reported emotion regulation in the DEF group, whereas the ABC group did not show a significant association. This finding may suggest that the atypical negative amygdala↔OFC functional coupling displayed in the DEF group may leads to emotion regulation difficulties in high-risk contexts. Of course, care must be taken in making such inferences due to the sample sizes in the intervention groups. The lack of significant association in the ABC group may indicate that this circuit is not yet functioning as a top-down regulatory mechanism during middle childhood. Instead, children in the ABC group may continue to capitalize on social buffering. Although we are limited in our ability to make causal inferences about change in children’s developmental trajectory due to our cross-sectional study design, our results suggest that the ABC intervention may causally shape the development of the emotion circuitry in children at risk for perturbed neurodevelopment in middle childhood.

The lateral OFC where the intervention effects were found plays a key role in fear learning, fear-related behavior regulation, and negative experience appraisal, such as non-reward and punishment. Given that children at high risk are subjected to interactions with their caregivers that are threatening and non-rewarding, suboptimal early care could result in changes to the function of the OFC and an increased likelihood of difficulties in regulating emotions later during their development. Although the literature is rife with inconsistent findings about the direction of functional connectivity in adversity-exposed populations, our findings are in line with Park and colleagues’ (2018) who found that more negative functional connectivity in young children is associated with difficulties in attentional functioning. In a group of young adults, a negative association was observed between early-life emotional abuse and rs-fc between amygdala and this region of the OFC (Fan et al., 2014). Depressed young adults also showed more left amygdala↔OFC anti-correlation than their non-depressed counterparts (Zhang et al., 2014), suggesting that atypical negative amygdala↔OFC rs-fc is a likely consequence of earlylife adversity and is associated with regulatory difficulties. Importantly, given the mixed results in the literature, more research is needed to replicate our findings.

The present study has several strengths and limitations. First, the study’s external validity is high because the families were recruited from a program designed to decrease foster care. Second, the longitudinal study design with randomization allows us to make prospective causal inferences about the observed effects. The study also has limitations, including the relatively short resting-state scan, limiting the signal-to-noise ratio of our BOLD measures. Furthermore, we only examined OFC, which stopped us from finding effects outside of this area. This was done because of our a priori focus on OFC and concerns about power due to our modest sample size. Moreover, the cross-sectional study design, along with the fact that not all randomized participants were considered in the final analyses, weakens the strength of drawing definitive causal conclusions from the current imaging data. This exclusion occurred because the participants did not take part in the fMRI sub-study, had excessive motion in the imaging data, or because they did not complete a successful prior EEG, which may limit the variability in the sample that participated in the MRI sub-study. Nonetheless, the similarity in the number of attritted participants across intervention groups suggests that these attritional factors may have impacted both groups comparably. However, it is crucial to acknowledge that the final sample of participants in the RCT was relatively small, making the study vulnerable to both Type 1 and Type 2 errors. The exploratory moderation analyses may be particularly vulnerable to Type 1 error, due to the small cell size in each group. Finally, we cannot make strong inferences about developmental processes, which would require longitudinal data. Our inferences about the nature of the neurodevelopmental process are speculative but are theoretically driven, as they build on prior longitudinal work (Gabard-Durnam et al., 2014; Qin et al., 2012). Alternatively, our result could indicate static differences in connectivity between the groups (rather than changes in neurodevelopment), but this interpretation is unlikely given the fact that rs-fc amygdala↔OFC rs-fc changes over time. These limitations underscore the importance of replicating the current findings in larger and different longitudinal samples.

Taken together, children show profound neurobiological adaptations to adverse developmental contexts, which may place them at increased risk for regulatory difficulties later in life (McLaughlin et al., 2019; VanTieghem et al., 2021). This makes it imperative to intervene early to help parents at risk for providing suboptimal care learn nurturing and responsive ways of parenting. Despite the limitations of this study, we demonstrated that the ABC intervention – a short, 10-session parenting program that enhances sensitive and responsive care – has long-lasting effects on vulnerable children’s amygdala↔OFC rs-fc coupling approximately 8 years after the families received the intervention. Multiple RCTs have demonstrated that ABC can enhance the quality of early care that children receive, which in turn improves children’s biobehavioral outcomes (Dozier & Bernard, 2019). Our findings add to this rich evidence base by showing that ABC has the potential to causally promote amygdala↔OFC rs-fc coupling during middle childhoods. The observed connectivity pattern in the ABC group resembles that seen in typically developing low-risk children and is significantly different from the amygdala↔OFC coupling of children in the DEF group who experienced early insensitive care without the benefits of ABC. Overall, these findings suggest a possible neural pathway through which early sensitive care may enhance healthy emotion regulation in children at risk for early insensitive care.

## Supplementary Material

1

## Figures and Tables

**Table 1.** Baseline demographic information across intervention groups at the post-intervention follow-up visit; taken from Bernard et al., 2012

Variable	Intervention group	
	
	ABC (*N* = 60)	DEF (*N* = 60)

Child male	62%	53%

Child belongs to a racial/ethnic minority group	93%	92%

Parent age	*M* = 29.0 years (*SD* = 7.3)	*M* = 29.0 years (*SD* = 8.7)

Parent belongs to a racial/ethnic minority group	78%	81%

**Table 2.** Sociodemographic characteristics and emotion regulation scores for children in the ABC and DEF groups

	ABC group (*n* = 21)		DEF group (*n* = 17)		Group differencet	
			
	*M*	*SD*	*M*	*SD*	t/ χ^2^	*p* value^a^

Sex, *N* (%)						

Female	10 (47.6%)		8 (47.06%)		χ2(1, *N* = 38) = 0	1^b^

Male	11 (52.4%)		9 (52.94%)			

Race, *N* (%)						

African American	13 (61.9%)		14 (82.35%)		χ2(3, *N* = 38) = 4.57	.206^b^

Biracial	6 (28.57%)		1 (5.88%)			

European American or White	1 (4.76%)		0 (0%)			

Hispanic or Latino/a	1 (4.76%)		2 (11.76%)			

Ethnicity, *N* (%)						

Hispanic or Latino/a	2 (9.52%)		3 (17.65%)		χ2(1, *N* = 38) = .065	.799^b^

Non-Hispanic or Non-Latino/a	19 (90.48%)		14 (82.35%)			

Age (years)						

Avg. age at MRI scan	10.13	.85	9.86	1.04	*t*(1, 36) = −.877	.387^b^

Age range at MRI scan	8.32–11.7		8.08–12.01			

Family income^c^						

Mean income at MRI scan	$27,296	$21,205	$25,074	$16,415	*t*(1, 36) = −.362	.719^b^

Income range at MRI scan	$1,903–$80K		$2,904–$55K			

Parental education	2.43	1.33	2.71	1.1	*t*(1, 36) = .690	.495^b^

Emotion regulation subscale	3.26	.46	3.32	.59	*t*(1, 36) = .169	.867^b^

Avg. time diff.: ERC–MRI	0.41 year	.678	0.493 year	.923	*t*(1, 36) = .318	.752^b^

Av. num. of usable volumes (range)	163 (109–176)	20	156 (92–176)	25.4	*t*(1, 36) = −.909	.369^b^

ABC = Attachment and Biobehavioral Catch-up; DEF = Developmental Education for Families.

a Significant at the *p* = .05 level, two-sided test.

b Educational background was measured on a scale of 6 (1 = did not complete high-school; 2 = GED; 3 = high-school diploma; 4 = some college; 5 = 4-year college degree; 6 = postgraduate degree (MA, MBA, PhD, JD, MD)).

c Four families did not report income information.

**Table 3.** Simple slopes for the interactions between amygdala↔OFC resting-state functional connectivity and intervention group predicting emotion regulation

	ABC group (*n* = 21)					DEF group (*n* = 17)					Interaction effect			
			
	Intercept	*b*	*SE*	*t*	*p*	Intercept	*b*	*SE*	*t*	*p*	*b*	*SE*	*t*	*p* value

ERC	3.992	−.172	.215	−.799	.43	4.328	.415	.186	2.231	.033	−.587	.278	−2.118	.042

ABC = Attachment and Biobehavioral Catch-up; DEF = Developmental Education for Families; ERC = Emotion Regulation Subscale of the Emotion Regulation Checklist. Covariates of no interest were age and sex.
