# Supplementary material for: Preliminary examination of the effects of an early parenting intervention on amygdala-orbitofrontal cortex resting-state functional connectivity among high-risk children: A randomized clinical trial
Source: Dev Psychopathol. Author manuscript; Available in PMC 2025 Aug 1. (PMC11260902; doi:10.1017/S0954579423001669)
Supplement: 1 [file NIHMS1954103-supplement-1.docx]

Supplementary Materials

**Referred** (*n* = 404)

**Not Consented** (*n* = 144)

♦  Unable to reach/Unresponsive (*n* = 79)

♦  Declined to participate (*n* = 32)

♦  Child placed out of home (*n* = 27)

♦  CG moved out of state (*n* = 4)

♦  Other (Staff Concern; *n* = 2)

**Enrolled in Intervention** (*n* = 212)

**Consented** (*n* = 260)

Excluded Pre-intervention (*n*= 48)

♦  Unable to reach/Unresponsive (*n* = 32)

♦  Declined to participate (*n* = 9)

♦  Child placed out of home (*n* = 6)

♦  CG moved out of state (*n* = 1)

**Assigned to ABC intervention** (*n* = 100)

♦ Completed 10 sessions (*n* = 78; 78% )

♦ Did not complete (*n* = 22)

♦  Unable to reach/Unresponsive (*n* = 17)

♦  Declined to participate (*n* = 4)

♦  Child placed out of home (*n* = 1)

**Assigned to DEF intervention** (*n* = 112)

♦ Completed 10 sessions (*n* = 92; 82% )

♦ Did not complete (*n* = 20)

♦  Unable to reach/Unresponsive (*n* = 17)

♦  Declined to participate (*n* = 1)

♦  Child placed out of home (*n* = 2)

**Participated in 8-year follow-up (*n* = 112)**

**8 year follow-up**

♦  Contacted (*n =* 58)

♦ Consented/Participated (*n* = 50)

♦ Did not participate/no show (*n* = 8)

**ABC Completers**

♦  Participated in follow-up (*n* = 78)

♦  Did not participate in follow-up (*n* = 0)

**ABC Non-completers**

♦  Participated in follow-up (*n* = 8)

♦  Did not participate in follow-up (*n* = 14)

**8 year EEG Sub-study**

♦ Did not complete EEG (*n* = 3)

♦ Only home portion of visit completed (*n* = 2)

♦ Developmental Issues (*n* = 1)

♦  Completed EEG (*n* = 47)

 ♦  Did not complete EEG (*n* = )

**8 year EEG Sub-study**

♦ Did not complete EEG (*n* = 4)

♦ Out of State (*n* = 2)

♦ Developmental Issues (*n* = 2)

♦  Completed EEG (*n* = 58)

 ♦  Did not complete EEG (*n* = )

**8 year follow-up**

♦  Contacted (*n* = 69)

♦  Consented/Participated (*n* = 62)

♦  Did not participate/no show (*n* = 7)

**Participated in follow-up during infancy or early childhood** (*n* = 183)

**DEF Completers**

♦  Participated in follow-up (*n* = 89)

♦  Did not participate in follow-up (*n* = 3)

♦  Child placed out of home (*n* = 3)

**DEF Non-completers**

♦  Participated in follow-up (*n* = 8)

♦  Did not participate in follow-up (*n* = 12)

**Current fMRI Sub-study Completion**

♦ Did not complete resting-state scan (*n* = 1)

♦ Completed resting-state scan (*n* = 26)

♦ Data included in analysis (*n* = 21)

♦ Data excluded from analysis (*n* = 5)

♦ Excessive motion (*n* = 3)

♦ Image registration or acquisition problems (*n* = 2)

**Current fMRI Sub-study Recruitment**

♦ Contacted (*n =* 34)

♦ Consented/Participated (*n* = 27)

♦ Did not participate/no show (*n* = 7)

**Current fMRI Sub-study Recruitment**

♦ Contacted (*n =* 31)

♦ Consented/Participated (*n* = 27)

♦ Did not participate/no show (*n* = 4)

**Current fMRI Sub-study Completion**

♦ Did not complete resting-state scan (*n* = 1)

♦ Completed resting-state scan (*n* = 26)

♦ Data included in analysis (*n* = 17)

♦ Data excluded from analysis (*n* = 9)

♦ Excessive motion (*n* = 8)

♦ Image registration or acquisition problems (*n* = 1)

**Information on data exclusion in the low-risk group:**

**Current fMRI Sub-study Completion**

♦ Did not complete resting-state scan (*n* = 0)

♦ Completed resting-state task (*n* = 26)

♦ Data included in analysis (*n* = 19)

♦ Data excluded from analysis (*n* = 7)

♦ Excessive motion (*n* = 2)

♦ Image registration or acquisition problems (*n* = 5)

***Supplementary Figure 1.*** CONSORT Study Flow Diagram for participants of randomized-clinical trial and present magnetic resonance imaging sub-study.


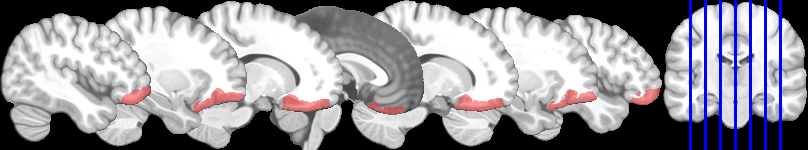


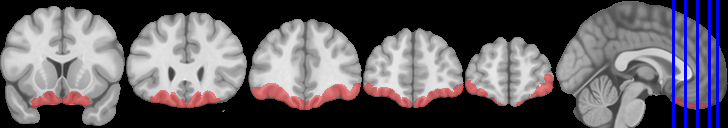


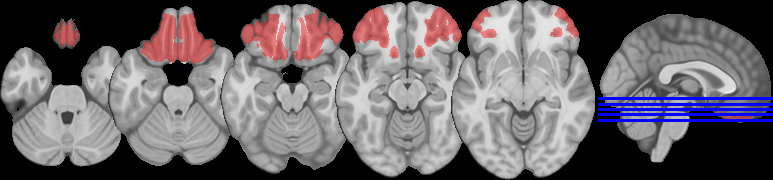


***Supplementary Figure 2***. OFC mask used in the present study. This mask was created using the Julich-Brain 3D probabilistic atlas. The mask is overlaid on the MNI 2009a atlas and denoted in pink. The blue li slices to the right of each row indicates (by blue lines) the location of each slice shown.


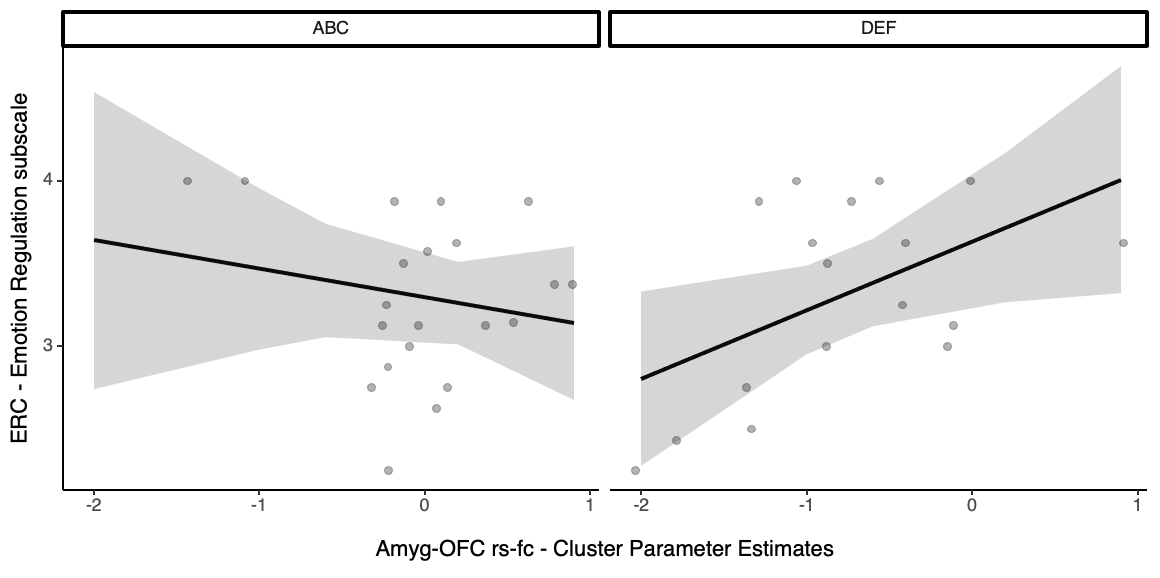


***Supplementary Figure 3***. Significant interaction effect. Intervention assignment interacts with amygdala-OFC rs-fc predicting children’s emotion regulation outcomes on the Emotion Regulation Checklist (Emotion Regulation subscale).

***Supplementary Table 1:*** Correlation matrix of study variables (N = 38)

|  | **1** | **2** | **3** | **4** | **5** | **6** | **7** |
| --- | --- | --- | --- | --- | --- | --- | --- |
| 1. **Age at MRI scan** | 1 |  |  |  |  |  |  |
| 1. **Usable volumes** | .209 | 1 |  |  |  |  |  |
| 1. **Functional connectivity** | -.006 | -.244 | 1 |  |  |  |  |
| 1. **DVARS** | -.324* | .245 | -.060 | 1 |  |  |  |
| 1. **SVARS** | -.425* | -.596*** | -.030 | .448** | 1 |  |  |
| 1. **DVARS^2^** | .026 | -.656*** | .081 | -.564*** | .236 | 1 |  |
| 1. **SVARS^2^** | .015 | -.676*** | .093 | -.584*** | .225 | .988*** | 1 |

*Note: DVARS - root mean square of the temporal change of the fMRI voxel-wise signal at each time point; SVARS - slow-wave variant of DVARS.*

** p < .05; * p < .01; *** p < .001*

|  | **COMP group (n = 19)** | |
| --- | --- | --- |
|  | **M** | **SD** |
| **Sex, No. (%)**  Female  Male | 9 (47.37%)  10 (52.63%) |  |
| **Race, No. (%)**  African American  Biracial  European American or White  Hispanic or Latino/a | 12 (63.16%)  1 (5.26%)  5 (26.32)  1 (5.26%) | |
| **Ethnicity, No. (%)**  Hispanic or Latino/a | 4 (21.05%) |  |
| Non-Hispanic or Non-Latino/a | 15 (78.95%) |  |
| **Age (years)** |  |  |
| Avg. age at MRI scan | 10.0 | .67 |
| Age range at MRI scan | 9.08 - 11 |  |
| **Family Income^c^** |  |  |
| Mean income at MRI scan | $ 50,120 | $30,115 |
| Income range at MRI scan | $20K – $120K |  |
| **Parental Education** | 3.68 | .82 |
| **Emotion Regulation Subscale** | 3.36 | .33 |
| **Avg. time diff.: ERC - MRI** | .543 year | .568 |
| **Av. num. of usable volumes (range)** | 154 (93 - 176) | 29.7 |

***Supplementary Table 2:*** Demographic information and descriptive neuroimaging statistics for the low-risk comparison groups.
